# (3*R*,8a*S*)-3-Ethyl­perhydro­pyrrolo[1,2-*a*]pyrazine-1,4-dione

**DOI:** 10.1107/S1600536810001856

**Published:** 2010-01-23

**Authors:** Adailton J. Bortoluzzi, Luciana B. P. Souza, Antonio C. Joussef

**Affiliations:** a Depto. de Química, Universidade Federal de Santa Catarina, 88040-900 Florianópolis, SC, Brazil

## Abstract

In the title compound, C_9_H_14_N_2_O_2_, the pyrrolidine and piperazine rings adopt envelope and boat conformations, respectively. The chiral centers were assigned on the basis of the known stereogenic center of an enanti­omerically pure starting material and the *trans* relationship between the H atoms attached to these centers. The crystal packing is stabilized by an inter­molecular hydrogen bond between the N—H group and a carbonyl O atom of the diketopiperazine group, forming zigzag *C*(5) chains along [010].

## Related literature

For general background to the chemistry and biological properties of diketopiperazines, see: Herbert & Kelleher (1994 ▶); Ciajolo *et al.* (1995 ▶); Morley *et al.* (1981 ▶); Kazuharu *et al.* (1990 ▶); Funabashi *et al.* (1994 ▶); Moyroud *et al.* (1996 ▶); Caballero *et al.* (2003 ▶); Onishi *et al.* (2003 ▶); Alberch *et al.* (2004 ▶); von Nussbaum *et al.* (2003 ▶). For related structures, see: Hendea *et al.* (2006 ▶).
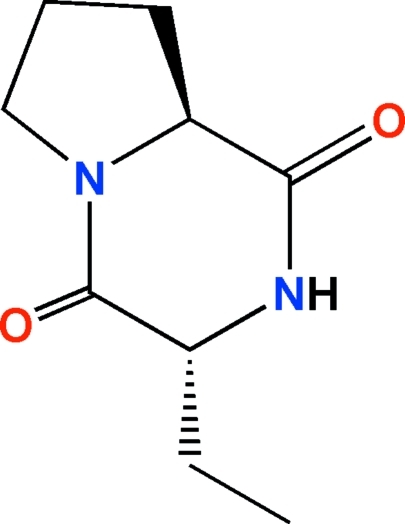


## Experimental

### 

#### Crystal data

C_9_H_14_N_2_O_2_
*M* *_r_* = 182.22
Monoclinic, 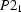

*a* = 6.8657 (4) Å
*b* = 9.9258 (17) Å
*c* = 7.0040 (5) Å
β = 90.892 (6)°
*V* = 477.25 (9) Å^3^
*Z* = 2
Mo *K*α radiation
μ = 0.09 mm^−1^
*T* = 293 K
0.46 × 0.40 × 0.33 mm

#### Data collection

Enraf–Nonius CAD-4 diffractometer
1290 measured reflections
1200 independent reflections
937 reflections with *I* > 2σ(*I*)
*R*_int_ = 0.032
3 standard reflections every 200 reflections  intensity decay: 1%

#### Refinement

*R*[*F* ^2^ > 2σ(*F* ^2^)] = 0.044
*wR*(*F* ^2^) = 0.120
*S* = 1.09
1200 reflections
119 parameters
1 restraint
H-atom parameters constrained
Δρ_max_ = 0.17 e Å^−3^
Δρ_min_ = −0.15 e Å^−3^

### 

Data collection: *CAD-4 Software* (Enraf–Nonius, 1989 ▶); cell refinement: *CAD-4 Software*; data reduction: *HELENA* (Spek, 1996 ▶); program(s) used to solve structure: *SIR97* (Altomare *et al.*, 1999 ▶); program(s) used to refine structure: *SHELXL97* (Sheldrick, 2008 ▶); molecular graphics: *PLATON* (Spek, 2009 ▶) and *Mercury* (Macrae *et al.*, 2006 ▶); software used to prepare material for publication: *SHELXL97*.

## Supplementary Material

Crystal structure: contains datablocks global, I. DOI: 10.1107/S1600536810001856/bh2268sup1.cif

Structure factors: contains datablocks I. DOI: 10.1107/S1600536810001856/bh2268Isup2.hkl

Additional supplementary materials:  crystallographic information; 3D view; checkCIF report

## Figures and Tables

**Table 1:** Hydrogen-bond geometry (Å, °)

*D*—H⋯*A*	*D*—H	H⋯*A*	*D*⋯*A*	*D*—H⋯*A*
N2—H2⋯O4^i^	0.87	1.98	2.817 (3)	161

Symmetry code: (i) 
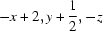
.

**Fig. 1.**
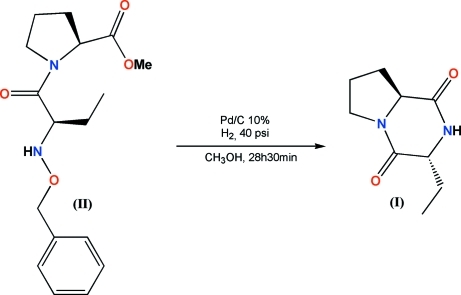
Synthetic route.

**Fig. 2.**
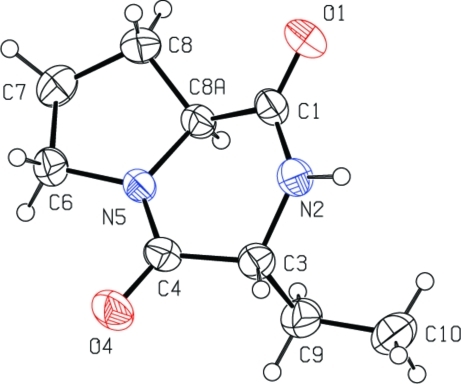
The molecular structure of the title compound with labeling scheme. Displacement ellipsoids are shown at the 40% probability level.

**Fig. 3.**
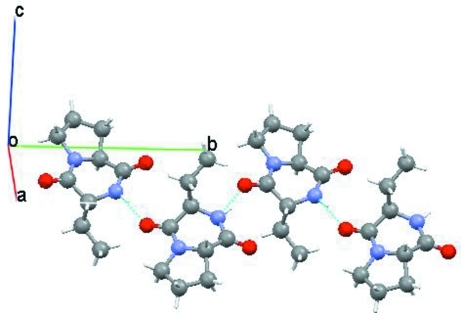
Polymeric chain along b axis formed by intermolecular hydrogen bonding. Symmetry code: -x + 2, y + 1/2, -z

**Fig. 4.**
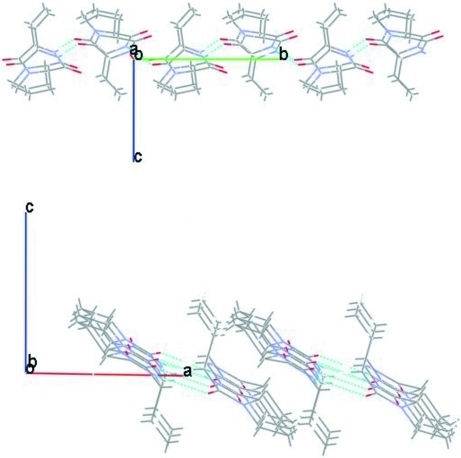
Partial packing of the title compound showing the stacking of the molecules along [100] (top) and along [010] (bottom).

## supplementary crystallographic information

### Comment

Diketopiperazine (DKP) backbone is an important pharmacophore in medicinal chemistry, which is conformationally restrained by six-membered ring with side chains that are oriented in a spatially defined manner (Herbert & Kelleher, 1994; Ciajolo *et al.*, 1995). DKPs are quite common in nature and many natural products with the DKP scaffold have been isolated encompassing a wide range of biological activities (Morley *et al.*, 1981; Kazuharu *et al.*, 1990; Funabashi *et al.*, 1994; Moyroud *et al.*, 1996). Several secondary metabolites of microorganisms with interesting biological properties contain a proline-derived diketopiperazine as part of their molecular skeleton (Caballero *et al.*, 2003; Onishi *et al.*, 2003; Alberch *et al.*, 2004; von Nussbaum *et al.*, 2003). During our work on the synthesis of L-proline-based DKPs, we prepared L-proline methyl ester derivative (II) which, under hydrogenolysis condition, led to the title compound (I, Fig. 1). Despite its full chemical characterization and the known configuration of the starting material L-proline, the absolute configuration at C3 was tentatively assigned as being *R* due to the *trans* relationship of the hydrogen atoms attached to carbons C3 and C8*a* based on ^1^H-NMR and NOE experiments. The crystallographic data unambiguously confirmed the *trans* relationship of the above mentioned hydrogen atoms and consequently the *R* configuration of the chiral center at C3 (Fig. 2).

The molecular structure of (I) consists of a bicycle system formed by pyrrolidine and piperazine fused rings (Hendea *et al.*, 2006). The five-membered pyrrolidine ring shows an envelope conformation, which is enveloped at C8. Piperazine ring shows perfect boat conformation, where N2, C1, C4 and N5 atoms lie on the basal plane (r.m.s. deviation: 0.0016 Å) and C3 and C8*a* are out of the basal mean plane by 0.39 Å (average) toward the same direction.

Strong intermolecular hydrogen bonds between the N—H group and the carbonyl O atom of the diketopiperazine neighboring groups contribute to the stabilization of the crystal structure. N2—H2···O4*^i^* [symmetry code: (*i*) -*x* + 2, *y* + 1/2, -*z*] interactions promote the formation of parallel one-dimensional zigzag *C*(5) chains running on the 2_1_ screw axis along [010] (Fig. 3). Furthermore, the molecules of (I) are stacked viewing in perpendicular projection of the chains, along [100], and viewing in parallel projection of the chains, along [010] (Fig. 4).

### Experimental

To a solution of L-proline methyl ester derivative (II) (0.084 mmol) in methanol (6 ml) was added Pd/C (10%, 18 mg, three portions of 6 mg of the catalyst were added each 10 h) and the mixture was shaken under 40 psi of hydrogen at room temperature for 28 h and 30 min [TLC control, alumina, ethyl acetate/hexane (1:3 *v*/*v*)]. After filtration of the catalyst, the solvent was evaporated under reduced pressure and column chromatography of the residue over alumina with ethyl acetate afforded compound (I) as a white solid, with 84% yield. A careful crystallization from ethyl acetate/hexane (1:3 *v*/*v*) provided crystals (mp. 133.5–134.5°C) suitable for X-ray analysis.

### Refinement

All non-H atoms were refined with anisotropic displacement parameters. H atoms were placed at their idealized positions with distances of 0.98, 0.97 and 0.96 Å for CH, CH_2_ and CH_3_, respectively. *U*_iso_ of the H atoms were fixed at 1.2 times for methine and methylene and 1.5 times for methyl of the *U*_eq_ of the carrier C atom. Hydrogen atom of the cyclic piperazine amine group was found in a difference map and treated with a riding model and its *U*_iso_ was also fixed at 1.2 times *U*_eq_ of the parent N atom.

### Crystal data

**Table:**

C_9_H_14_N_2_O_2_	*F*(000) = 196
*M**_r_* = 182.22	*D*_x_ = 1.268 Mg m^−^^3^
Monoclinic, *P*2_1_	Melting point: 407 K
Hall symbol: P 2yb	Mo *K*α radiation, λ = 0.71073 Å
*a* = 6.8657 (4) Å	Cell parameters from 25 reflections
*b* = 9.9258 (17) Å	θ = 3.6–15.6°
*c* = 7.0040 (5) Å	µ = 0.09 mm^−^^1^
β = 90.892 (6)°	*T* = 293 K
*V* = 477.25 (9) Å^3^	Prism, colorless
*Z* = 2	0.46 × 0.40 × 0.33 mm

### Data collection

**Table:**

Enraf–Nonius CAD-4 diffractometer	*R*_int_ = 0.032
Radiation source: fine-focus sealed tube	θ_max_ = 27.9°, θ_min_ = 3.0°
graphite	*h* = −9→9
ω–2θ scans	*k* = −13→0
1290 measured reflections	*l* = −9→0
1200 independent reflections	3 standard reflections every 200 reflections
937 reflections with *I* > 2σ(*I*)	intensity decay: 1%

### Refinement

**Table:**

Refinement on *F*^2^	Primary atom site location: structure-invariant direct methods
Least-squares matrix: full	Secondary atom site location: difference Fourier map
*R*[*F*^2^ > 2σ(*F*^2^)] = 0.044	Hydrogen site location: inferred from neighbouring sites
*wR*(*F*^2^) = 0.120	H-atom parameters constrained
*S* = 1.09	*w* = 1/[σ^2^(*F*_o_^2^) + (0.0525*P*)^2^ + 0.0801*P*] where *P* = (*F*_o_^2^ + 2*F*_c_^2^)/3
1200 reflections	(Δ/σ)_max_ < 0.001
119 parameters	Δρ_max_ = 0.17 e Å^−^^3^
1 restraint	Δρ_min_ = −0.15 e Å^−^^3^
0 constraints	

### Fractional atomic coordinates and isotropic or equivalent isotropic displacement parameters (Å^2^)

**Table:**

	*x*	*y*	*z*	*U*_iso_*/*U*_eq_	
C1	0.6671 (5)	0.5429 (3)	0.1100 (4)	0.0446 (7)	
C3	0.8672 (4)	0.3662 (3)	−0.0490 (4)	0.0446 (7)	
H3	1.0083	0.3519	−0.0400	0.054*	
C4	0.7726 (4)	0.2617 (3)	0.0781 (4)	0.0428 (7)	
C6	0.5113 (5)	0.2151 (3)	0.3021 (5)	0.0481 (7)	
H6A	0.5912	0.1966	0.4145	0.058*	
H6B	0.4732	0.1304	0.2435	0.058*	
C7	0.3339 (5)	0.2985 (4)	0.3527 (5)	0.0646 (10)	
H7A	0.3015	0.2867	0.4860	0.077*	
H7B	0.2223	0.2732	0.2741	0.077*	
C8	0.3944 (5)	0.4437 (4)	0.3131 (5)	0.0603 (9)	
H8A	0.2816	0.5001	0.2878	0.072*	
H8B	0.4681	0.4810	0.4199	0.072*	
C8A	0.5200 (4)	0.4315 (3)	0.1373 (4)	0.0415 (6)	
H8AA	0.4353	0.4263	0.0237	0.050*	
C9	0.8066 (5)	0.3439 (4)	−0.2576 (5)	0.0572 (9)	
H9A	0.8351	0.2514	−0.2924	0.069*	
H9B	0.6670	0.3568	−0.2705	0.069*	
C10	0.9082 (6)	0.4374 (5)	−0.3946 (5)	0.0691 (11)	
H10A	1.0466	0.4307	−0.3753	0.104*	
H10B	0.8673	0.5284	−0.3720	0.104*	
H10C	0.8751	0.4124	−0.5235	0.104*	
N2	0.8290 (4)	0.5027 (2)	0.0198 (4)	0.0486 (7)	
H2	0.9129	0.5629	−0.0171	0.058*	
N5	0.6154 (3)	0.3007 (2)	0.1667 (3)	0.0396 (6)	
O1	0.6391 (4)	0.6573 (2)	0.1630 (4)	0.0643 (7)	
O4	0.8399 (4)	0.1481 (2)	0.0937 (4)	0.0655 (7)	

### Atomic displacement parameters (Å^2^)

**Table:**

	*U*^11^	*U*^22^	*U*^33^	*U*^12^	*U*^13^	*U*^23^
C1	0.0549 (17)	0.0338 (15)	0.0452 (15)	0.0027 (13)	−0.0003 (13)	0.0037 (13)
C3	0.0380 (15)	0.0432 (17)	0.0528 (16)	0.0016 (12)	0.0064 (13)	0.0008 (14)
C4	0.0405 (15)	0.0384 (15)	0.0494 (16)	0.0032 (13)	0.0012 (13)	−0.0032 (13)
C6	0.0556 (18)	0.0402 (16)	0.0486 (16)	−0.0063 (14)	0.0040 (14)	0.0026 (14)
C7	0.061 (2)	0.067 (2)	0.066 (2)	0.0009 (19)	0.0250 (16)	0.005 (2)
C8	0.065 (2)	0.050 (2)	0.067 (2)	0.0113 (17)	0.0253 (16)	0.0011 (17)
C8A	0.0415 (14)	0.0351 (14)	0.0480 (15)	0.0053 (13)	0.0043 (12)	0.0018 (12)
C9	0.066 (2)	0.055 (2)	0.0507 (17)	−0.0062 (16)	0.0063 (16)	−0.0058 (15)
C10	0.077 (2)	0.079 (3)	0.0526 (19)	−0.003 (2)	0.0156 (17)	−0.0057 (19)
N2	0.0534 (15)	0.0378 (14)	0.0549 (15)	−0.0127 (12)	0.0085 (12)	−0.0031 (11)
N5	0.0416 (13)	0.0317 (12)	0.0457 (13)	0.0024 (10)	0.0051 (10)	0.0018 (11)
O1	0.0847 (18)	0.0346 (12)	0.0737 (16)	0.0033 (12)	0.0073 (14)	−0.0050 (12)
O4	0.0627 (14)	0.0468 (14)	0.0875 (17)	0.0208 (12)	0.0180 (12)	0.0095 (13)

### Geometric parameters (Å, °)

**Table:**

C1—O1	1.210 (4)	C7—H7A	0.9700
C1—N2	1.347 (4)	C7—H7B	0.9700
C1—C8A	1.512 (4)	C8—C8A	1.519 (4)
C3—N2	1.464 (4)	C8—H8A	0.9700
C3—C4	1.519 (4)	C8—H8B	0.9700
C3—C9	1.529 (4)	C8A—N5	1.468 (4)
C3—H3	0.9800	C8A—H8AA	0.9800
C4—O4	1.223 (4)	C9—C10	1.513 (5)
C4—N5	1.312 (4)	C9—H9A	0.9700
C6—N5	1.467 (4)	C9—H9B	0.9700
C6—C7	1.519 (5)	C10—H10A	0.9600
C6—H6A	0.9700	C10—H10B	0.9600
C6—H6B	0.9700	C10—H10C	0.9600
C7—C8	1.527 (6)	N2—H2	0.8717
			
O1—C1—N2	123.9 (3)	C8A—C8—H8B	111.1
O1—C1—C8A	122.5 (3)	C7—C8—H8B	111.1
N2—C1—C8A	113.6 (3)	H8A—C8—H8B	109.0
N2—C3—C4	111.0 (2)	N5—C8A—C1	111.6 (2)
N2—C3—C9	113.7 (3)	N5—C8A—C8	102.4 (2)
C4—C3—C9	110.4 (3)	C1—C8A—C8	115.7 (3)
N2—C3—H3	107.1	N5—C8A—H8AA	109.0
C4—C3—H3	107.1	C1—C8A—H8AA	109.0
C9—C3—H3	107.1	C8—C8A—H8AA	109.0
O4—C4—N5	122.8 (3)	C10—C9—C3	113.3 (3)
O4—C4—C3	121.2 (3)	C10—C9—H9A	108.9
N5—C4—C3	116.0 (3)	C3—C9—H9A	108.9
N5—C6—C7	103.6 (3)	C10—C9—H9B	108.9
N5—C6—H6A	111.0	C3—C9—H9B	108.9
C7—C6—H6A	111.0	H9A—C9—H9B	107.7
N5—C6—H6B	111.0	C9—C10—H10A	109.5
C7—C6—H6B	111.0	C9—C10—H10B	109.5
H6A—C6—H6B	109.0	H10A—C10—H10B	109.5
C6—C7—C8	104.5 (3)	C9—C10—H10C	109.5
C6—C7—H7A	110.8	H10A—C10—H10C	109.5
C8—C7—H7A	110.8	H10B—C10—H10C	109.5
C6—C7—H7B	110.8	C1—N2—C3	125.6 (3)
C8—C7—H7B	110.8	C1—N2—H2	119.3
H7A—C7—H7B	108.9	C3—N2—H2	114.5
C8A—C8—C7	103.4 (3)	C4—N5—C6	123.2 (3)
C8A—C8—H8A	111.1	C4—N5—C8A	124.3 (2)
C7—C8—H8A	111.1	C6—N5—C8A	112.5 (2)
			
N2—C3—C4—O4	−152.4 (3)	O1—C1—N2—C3	−179.1 (3)
C9—C3—C4—O4	80.6 (4)	C8A—C1—N2—C3	0.2 (4)
N2—C3—C4—N5	28.2 (4)	C4—C3—N2—C1	−31.8 (4)
C9—C3—C4—N5	−98.8 (3)	C9—C3—N2—C1	93.3 (3)
N5—C6—C7—C8	−23.9 (4)	O4—C4—N5—C6	3.8 (5)
C6—C7—C8—C8A	36.4 (4)	C3—C4—N5—C6	−176.8 (3)
O1—C1—C8A—N5	−147.5 (3)	O4—C4—N5—C8A	−173.9 (3)
N2—C1—C8A—N5	33.2 (3)	C3—C4—N5—C8A	5.4 (4)
O1—C1—C8A—C8	−31.1 (4)	C7—C6—N5—C4	−175.5 (3)
N2—C1—C8A—C8	149.7 (3)	C7—C6—N5—C8A	2.5 (3)
C7—C8—C8A—N5	−33.9 (3)	C1—C8A—N5—C4	−37.8 (4)
C7—C8—C8A—C1	−155.4 (3)	C8—C8A—N5—C4	−162.1 (3)
N2—C3—C9—C10	59.2 (4)	C1—C8A—N5—C6	144.3 (3)
C4—C3—C9—C10	−175.3 (3)	C8—C8A—N5—C6	20.0 (3)

### Hydrogen-bond geometry (Å, °)

**Table:**

*D*—H···*A*	*D*—H	H···*A*	*D*···*A*	*D*—H···*A*
N2—H2···O4^i^	0.87	1.98	2.817 (3)	161

Symmetry codes: (i) −*x*+2, *y*+1/2, −*z*.
